# MS-Helios: a Circos wrapper to visualize multi-omic datasets

**DOI:** 10.1186/s12859-018-2564-9

**Published:** 2019-01-11

**Authors:** Harald Marx, Joshua J. Coon

**Affiliations:** 1 Department of Microbiology and Ecosystems Science, University of Vienna, 1090 Vienna, Austria; 2 Department of Chemistry, University of Wisconsin–Madison, Madison, WI 53706 USA; 3 Morgridge Institute for Research, Madison, WI 53715 USA; 4 Genome Center of Wisconsin, Madison, WI 53706 USA; 5 Department of Biomolecular Chemistry, University of Wisconsin–Madison, Madison, WI 53706 USA

## Abstract

**Background:**

Advances in high-resolution mass spectrometry facilitate the identification of hundreds of metabolites, thousands of proteins and their post-translational modifications. This remarkable progress poses a challenge to data analysis and visualization, requiring methods to reduce dimensionality and represent the data in a compact way. To provide a more holistic view, we recently introduced circular proteome maps (CPMs). However, the CPM construction requires prior data transformation and extensive knowledge of the Perl-based tool, Circos.

**Results:**

We present MS-Helios, an easy to use command line tool with multiple built-in data processing functions, allowing non-expert users to construct CPMs or in general terms circular plots with a non-genomic basis. MS-Helios automatically generates data and configuration files to create high quality and publishable circular plots with Circos. We showcase the software on large-scale multi-omic datasets to visualize global trends and/or to contextualize specific features.

**Conclusions:**

MS-Helios provides the means to easily map and visualize multi-omic data in a comprehensive way. The software, datasets, source code, and tutorial are available at https://sourceforge.net/projects/ms-helios/.

## Background

Innovative high-throughput technologies, such as microarrays, next-generation sequencing, and mass-spectrometry (MS) have greatly advanced our understanding of biological systems. With these readily available, cost-effective, and comprehensive data acquisition methods, systems biology is undergoing a transition from single-omic to multi-omic data analysis [1]. However, integrating and visualizing thousands of multi-omic molecular profiles poses new challenges to systems biology. To date most multi-omic analysis methods rely on clustering, correlation [2], or dimensionality reduction methods, e.g., principal component analysis to transform the data prior to visualization [3].

To provide a holistic and integrated view, we recently introduced circular proteome maps (CPMs), visualizing sample features in a circular plot in a proteome-centric way [4]. Circular plots allow one to visualize high-dimensional data and feature relationships in an intuitive and aesthetic way, relying on well-known plot types, e.g., histograms, scatter plots, and line plots [5, 6]. In addition, data tracks provide the means to contextualize specific features over multiple omic levels. The gold standard software to build circular plots is Circos, a command line based Perl program with a steep learning curve [5]. Multiple R packages and tools are available to ease the construction process and visualization of circular plots [6–10]. These tools are either built for genomic data or map other data sources to a genomic basis; none of them consider multi-omic data integration or visualization with a non-genomic basis.

To ease the construction of circular plots with a non-genomic basis, we developed a Circos wrapper termed MS-Helios. MS-Helios is a command line tool that allows for fast prototyping, data exploration, and easy generation of high quality and publish-ready figures.

## Implementation

MS-Helios is a Java (1.8.0_121) desktop application with a command line interface (CLI). The CLI is built with the Apache Commons CLI library (1.3.1) to support GNU and POSIX like option syntax. MS-Helios and Circos (≥ 0.67–5) default parameters are set in Java property files. The built-in normalization and transformation methods use the Apache Commons Mathematics library (3.3.6.1).

To read an input file MS-Helios supports multiple field delimiters, e.g., comma, tabular, and space, as a regular expression. Input files have to be in a data matrix format, i.e. first row containing the sample names and first column the feature identifier. The first dataset defines ideogram order and initial feature coordinates in the stepwise construction, whereas subsequent datasets are data tracks. Each ideogram represents a sample and the respective end coordinates the sample size. MS-Helios provides various built-in functions to cluster, transform, normalize, sort and filter the input data. A naïve algorithm clusters ideogram features by sample occurrence. Cluster segments can be highlighted by Circos brewer colors and/or grid lines. To assign a sample specificity score to a feature, we implemented Shannon entropy [4], which is associated to the sample with the highest value. Sample-wise normalization methods include z-score, scaling [0, 1], and divide by min, max, mean, standard deviation or sum. Each data track can be sorted in ascending and descending order, to restructure the ideograms and respective data tracks. To highlight specific features MS-Helios supports a top-hat and percentile filter over samples by setting a threshold in the Circos rules configuration.

MS-Helios supports several Circos data track plot types, including histogram, scatter, line plot and wedge highlights. The Circos configuration is specific to each plot type, parameters are set for optimal visualization of large-scale data. To ease graphical post-processing of Circos plots, MS-Helios allows to partition the output by sample. Each construction step is stored in the MS-Helios file by serialization. MS-Helios writes Circos configuration and data files, as well as mapping files into an output folder.

## Results

### MS-Helios workflow

**Fig. 1:**
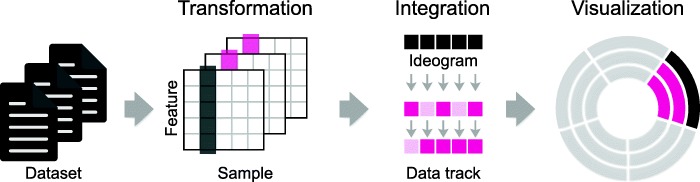
MS-Helios workflow. The construction of a circular proteome map (CPM) is a multi-step process. First, MS-Helios reads the datasets as data matrices, where the first row are sample names and the first column feature identifiers. Second, the data is processed with built-in transformation and normalization functions. Third, datasets are mapped to each other as ideogram and data tracks (light pink – missing values). In a final step, MS-Helios writes data and configuration files to construct a circular plot with Circos

MS-Helios builds circular plots with a non-genomic basis from datasets in delimited text file format, where rows represent features and columns samples (Fig. 1). To preprocess raw input data, MS-Helios supports a multitude of built-in normalization and transformation methods. Next, datasets are mapped stepwise to each other with common features and samples. The first dataset determines the initial order in the ideogram and subsequent datasets in the data tracks. In the final step, MS-Helios writes Circos data and configuration files. MS-Helios requires, for most use cases, almost no time to do the transformation and integration, allowing for fast prototyping. The plot construction with Circos may take more time depending on the number of data points. The default configuration of MS-Helios and Circos enables users to produce high quality and publishable figures, requiring minimal input from the user to build the data and config files for Circos. We showcase MS-Helios on a multi-omic *Sus scrofa* dataset [4, 11].

### Protein and transcript expression in juvenile *Sus scrofa* organs

**Fig. 2:**
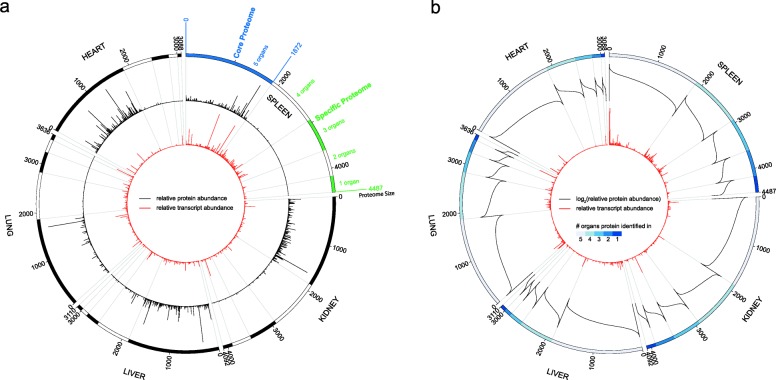
*Sus scrofa* circular proteome map. Circular proteome map (CPM) of protein and transcript abundance in five *Sus scrofa* organs (annotation and legends added post-construction). Ideograms represent organ proteomes and comprise protein clusters (grey grid lines). The first ideogram cluster highlights the core proteome. Black histograms are scaled (**a**) or sorted log2 transformed (**b**) protein abundance and red histograms are scaled transcript abundance

To exemplify a circular proteome map (CPM), we utilize *Sus scrofa* protein and transcript expression profiles of five organs. In Fig. 2 the ideograms represent organ proteomes and bars are clusters. Each ideogram cluster illustrates proteins by organ occurrence, e.g., the first cluster (Fig. 2a, blue bar) contains 1872 proteins present in five organs known as core proteome. To explore protein expression in the core and specific proteome (Fig. 2a, green bars), we utilize the built-in scaling normalization method (Fig. 2a, black histogram). The comparison reveals high abundance in the core for the most biological pervasive proteins, in contrast to the low abundant more specialized proteins. By mapping the transcript data to the proteomes (red histogram), we are able to illustrate similar trends in the core but the opposite for specific clusters. Each individual cluster illustrates that high abundant proteins correlate with high abundant transcripts, but this trend is not generalizable for the complete cluster (Fig. 2b).

## Conclusion

MS-Helios enables users to build circular plots with a non-genomic basis for exploration of high-dimensional multi-omic data without requiring any prior knowledge with Circos. MS-Helios implements the most useful Circos plot types, but also facilitates easy extension to other plot types. Our datasets demonstrate the aesthetics and power of circular plots to highlight intra and inter sample variation in feature abundance.

## Abbreviations

CLI: command line interface
CPM: circular proteome map
MS: Mass spectrometry
